# Extraction and Determination of Vitamin K_1_ in Foods by Ultrasound-Assisted Extraction, SPE, and LC-MS/MS

**DOI:** 10.3390/molecules25040839

**Published:** 2020-02-14

**Authors:** Yueqing Xu, Liangxiao Zhang, Ruinan Yang, Xu Yu, Li Yu, Fei Ma, Hui Li, Xiupin Wang, Peiwu Li

**Affiliations:** 1 Oil Crops Research Institute, Chinese Academy of Agricultural Sciences, Wuhan 430062, China; xuyueqing0710@126.com (Y.X.); yanrinannan@126.com (R.Y.); 13126832675@163.com (X.Y.); yuli01@caas.cn (L.Y.); mafei01@caas.cn (F.M.); lihui-gf@163.com (H.L.); wangxiupin01@caas.cn (X.W.); peiwuli@oilcrops.cn (P.L.); 2 Key Laboratory of Biology and Genetic Improvement of Oil Crops, Ministry of Agriculture and Rural Affairs, Wuhan 430062, China; 3 Laboratory of Quality and Safety Risk Assessment for Oilseed Products (Wuhan), Ministry of Agriculture and Rural Affairs, Wuhan 430062, China; 4 Quality Inspection and Test Center for Oilseed Products, Ministry of Agriculture and Rural Affairs, Wuhan 430062, China; 5 Key Laboratory of Detection for Mycotoxins, Ministry of Agriculture and Rural Affairs, Wuhan 430062, China

**Keywords:** ultrasound-assisted extraction, solid-phase extraction, LC-MS/MS, vitamin K_1_, foods

## Abstract

Vitamin K_1_ is one of the important hydrophobic vitamins in fat-containing foods. Traditionally, lipase is employed in the determination of vitamin K_1_ to remove the lipids, which makes the detection complex, time-consuming, and insensitive. In this study, the determination of vitamin K_1_ in fat-containing foods was developed based on ultrasound-assisted extraction (UAE), solid-phase extraction (SPE) combined with liquid chromatography–tandem mass spectrometry (LC-MS/MS). The optimal conditions for extraction of vitamin K_1_ were material–liquid ratio of 1:70 (g/mL), extraction temperature of 50 °C, extraction power of 700 W, extraction time of 50 min, material-wash fluid ratio of 1:60 (g/mL), and 8 mL of hexane/anhydrous ether (97:3, *v*/*v*) as the elution solvent. Then, vitamin K_1_ was analyzed on a ZORBAX SB-C18 column (50 mm × 2.1 mm, 1.8 μm) by gradient elution with water (0.01% formic acid) and methanol (0.01 formic acid + 2.5 mmol/L ammonium formate) as the mobile phase. The limit of detection (LOD) and limit of quantification (LOQ) were 0.05 and 0.16 μg/kg, respectively. Calibration curve was linear over the range of 10–500 ng/mL (*R*^2^ > 0.9988). The recoveries at three spiked levels were between 80.9% and 119.1%. The validation and application indicated that the proposed method was simple and sensitive in determination of vitamin K_1_ in fat-containing foods.

## 1. Introduction

Vitamin K is an essential dietary micronutrient. Vitamin K_1_ definitely accounts for more than 80% of the total vitamin K in the human diet, and most of our present knowledge on vitamin K concerns K_1_ [1]. Vitamin K_1_ is a fat-soluble, antihemorrhagic vitamin that is synthesized by plants, green algae, and some species of cyanobacteria. It belongs to a class of naphthoquinone derivatives with biological activity in chlorophyll [2,3]. Vitamin K_1_ is stable to air and moisture, but it is sensitive to light and alkaline conditions [4]. Vitamin K_1_ is essential for the formation of liver zymogen and can regulate and control the synthesis of coagulation factors II, VII, IX, and X. Vitamin K_1_ is used to treat bleeding disorders [5]. Moreover, it can also inhibit cancer [6], prevent vascular calcification [7], participate in bone metabolism [8], prevention of chronic kidney disease [9], and help treat pertussis syndrome [10]. In the human diet, green leafy vegetables and fat-containing foods, such as soybean, edible oil, and dairy products, supply vitamin K_1_ for human beings [11,12]. The adult consumes 1–2 μg of vitamin K_1_ per kilogram of body weight from food every day. Since there is no bacterium in the baby’s intestine to synthesize vitamin K_2_, it is recommended to consume 2 μg per kilogram of body weight. In addition, our assessment of vitamin K_1_ intake in the diet is incomplete [13]. There is increasing need for more reliable data on the vitamin K_1_ content of foods [14].

Since fat-containing foods contain hydrophobic components such as triglycerides that can be coextracted, it is more difficult to extract vitamin K_1_ from fat-containing foods than fruits and vegetables. Therefore, a complex pretreatment step is necessary to extract and purify vitamin K_1_ before further analysis [12]. The traditional pretreatment methods for vitamin K_1_ mainly include the saponification [15], enzymatic hydrolysis [16], and liquid-liquid extraction [1]. However, since vitamin K_1_ is easily decomposed by alkali, determination of vitamin K_1_ by the saponification method is inaccurate [17]. Moreover, enzymatic hydrolysis involves enzymatic hydrolysis with lipase, saponification with an alkali, and extraction with an organic solvent. Because of complex procedures, the recovery rate is usually low and the precision of detection result is poor. Meanwhile, liquid–liquid extraction requires a large amount of time and solvent, and the extraction efficiency is therefore low. Therefore, it is necessary to develop a new method to extract VK_1_ from fat-containing foods. Solid-phase extraction (SPE) is a sample pretreatment technology that has the advantages of high recovery rate and convenient operation [18,19]. Moreover, ultrasound-assisted extraction (UAE) is commonly used in sample preparation of analytical chemistry [20], which is cheap, efficient, adjustable, applicable, and easy to operate. The previous study indicated that UAE can improve the extraction efficiency of many target compounds [21]. Therefore, ultrasound-assisted extraction followed by solid-phase extraction might be good choice to extract and purify the vitamin K_1_ from fat-containing foods.

Generally, vitamin K_1_ was detected by fluorescence detection (FD) [22], electrochemical detection (ECD) [23], photodiode array (PDA) [24], ultraviolet detection (UV) [25], chemiluminescence (CL) [25], and mass spectrometry (MS or MS/MS) [26]. Liquid chromatography tandem mass spectrometry (LC-MS/MS) is a mature quantitative method used to analyze components of natural products. It can distinguish or eliminate the interference of other substances in a complex matrix with fast analysis speed, high sensitivity, and accurate quantitation [27,28,29].

In this study, to overcome the shortages of traditional extraction and detection methods on vitamin K_1_ in fat-containing foods, the ultrasound-assisted extraction and SPE combined with LC-MS/MS were used to determine the vitamin K_1_ content in fat-containing foods. This approach was easy to conduct and was effective at enrichment and purification of the samples. To validate the developed method, the contents of vitamin K_1_ in fat-containing foods such as oil seeds and edible oil were detected.

## 2. Results and Discussion

### 2.1. Optimization of Ultrasound-Assisted Extraction Conditions

In order to improve the efficiency of ultrasound-assisted extraction of vitamin K_1_, the parameters, including the material-liquid ratio, ultrasonic time, ultrasonic power, and ultrasonic temperature, were optimized. The common conditions selected were material–liquid ratio of 1/60 g/mL, ultrasonic time of 30 min, ultrasonic power of 700 W, ultrasonic temperature of 50 °C, material-wash fluid ratio of 1/60 g/mL, and elution solution ratio 97:3 (n-hexane : anhydrous ether, *v*/*v*).

#### 2.1.1. Effect of Material-Liquid Ratio

**Figure 1:**
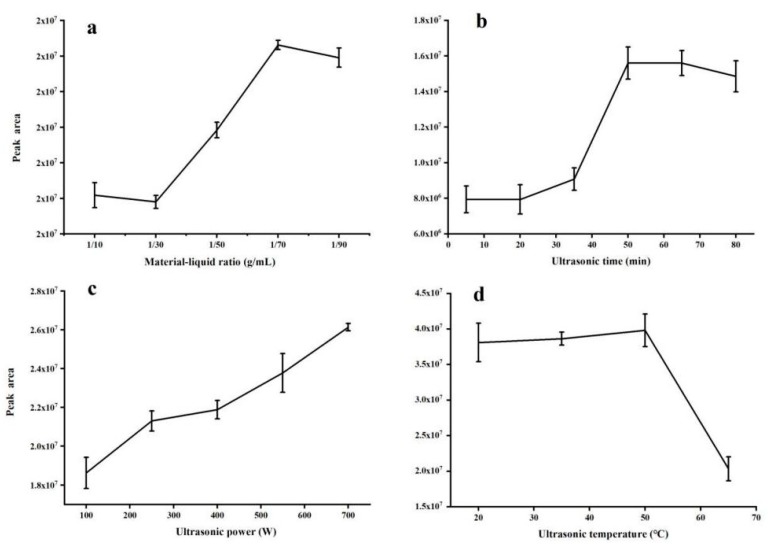
Influence of four parameters on extraction efficiency: (**a**) material-liquid ratio, (**b**) ultrasonic time, (**c**) ultrasonic power, and (**d**) ultrasonic temperature.

The effect of different material–liquid ratios (1/10, 1/30, 1/50, 1/70, and 1/90 g/mL) on compound extraction efficiency is shown in Figure 1a. When more n-hexane extract was added, the peak area of vitamin K_1_ reached a steady state when the liquid-to-liquid ratio was 1:70 (g/mL). A ratio of 1:70 (g/mL) gave the highest total yield. The optimal material-liquid ratio was determined to be 1:70 (g/mL).

#### 2.1.2. Effect of Ultrasonic Time

The effect of ultrasonic time on extraction efficiency was studied by varying the sonication time from 5 to 80 min. At 50 min, the peak area of vitamin K_1_ reached its maximum and then stabilized (Figure 1b). This may be because vitamin K_1_ is stable to air and moisture, and the longer the ultrasound time, the higher the extraction efficiency. The highest efficiency and optimal extraction time occurred at 50 min. 

#### 2.1.3. Effect of Ultrasonic Power

Ultrasonic power is an important factor in the ultrasonic extraction process. A DTC-27J ultrasonic water bath was used for the extraction experiment. This system has a fixed frequency of 40 kHz, but the power is adjustable (maximum power is 700 W). The target compound was extracted at 100, 250, 400, 550, and 700 W. As the ultrasonic power increased, the peak area of vitamin K_1_ and extraction efficiency also increased (Figure 1c). The ultrasonic power affects the cell wall of the ultrasonic fractured sample, which affects the dissolution of vitamin K_1_ into the extraction solvent. The ultrasonic power also affects the molecular motion efficiency of the extraction solvent, thereby affecting the contact, mutual fusion, and mixing rate of the extraction solvent with the vitamin K_1_ in the sample. In this study, the maximum power used for the extraction of the target compound was 700 W. 

#### 2.1.4. Effect of Ultrasonic Temperature

Ultrasonic time can influence compound solubility and stability. Vitamin K_1_ was extracted in an ultrasonic water bath at constant temperatures of 20, 35, 50, and 65 °C. As the extraction temperature increased, the extraction rate of vitamin K_1_ also increased (Figure 1d). When the temperature was 50 °C, the extraction rate of vitamin K_1_ was highest. A temperature increase beyond 50 °C reduced the extraction rate of vitamin K_1_. Therefore, 50 °C was chosen as the optimum ultrasonic extraction temperature.

### 2.2. Optimization of SPE Conditions

To improve the purification efficiency of solid-phase extraction column for vitamin K_1_, the parameters, including the material-wash fluid ratio and the elution solution ratio, were optimized. The common conditions selected were material–liquid ratio of 1/60 (g/mL), ultrasonic time of 30 min, ultrasonic power of 700 W, ultrasonic temperature of 50 °C, material-wash fluid ratio of 1/60 (g/mL), and elution solution ratio 97:3 (n-hexane : anhydrous ether, *v*/*v*).

#### 2.2.1. Effect of Material-Wash Fluid Ratio

**Figure 2:**
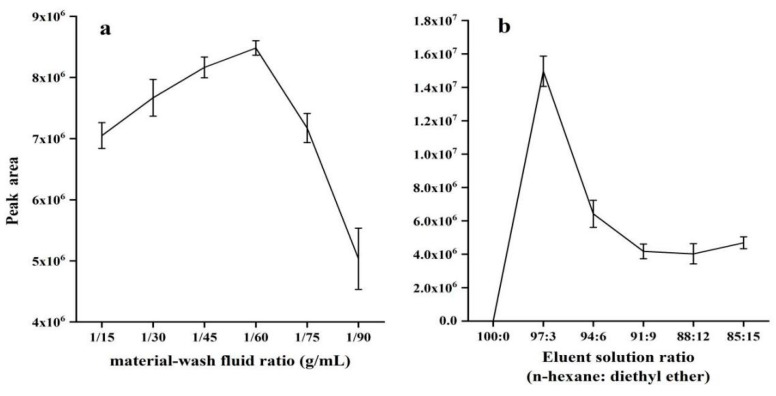
Influence of two parameters on Purification efficiency: (**a**) material-wash fluid ratio and (**b**) elution solution ratio.

Different material-wash fluid ratios (1/15, 1/30, 1/45, 1/60, 1/75, and 1/90 g/mL) were studied. The effects of the material-wash fluid ratios on the extraction efficiency of vitamin K_1_ are shown in Figure 2a. Extraction efficiency increased as volume of wash solvent was increased, but the extraction efficiency was highest when the material-wash fluid ratios were 1:60 (g/mL). However, extraction efficiency decreased after 1:60 (g/mL). A ratio of 1:60 (g/mL) generated the largest peak area and was therefore used for all subsequent work.

#### 2.2.2. Effect of Elution Solution Ratio

Because of the difference in the elution ability of n-hexane and anhydrous ether, a mixed solution of n-hexane and anhydrous ether was used as the eluent. The ratio of the two solvents was optimized. An 8 mL volume of n-hexane/anhydrous ether series solution (100:0, 97:3, 94:6, 91:9, 88:12, 85:15, *v*/*v*) was eluted into a 10 mL test tube. The peak area was highest and elution efficiency was best when the elution solution ratio was 97:3 (Figure 2b). As the proportion of anhydrous ether increased, more impurities were eluted, and the matrix effects increased, reducing the sensitivity. In this study, n-hexane/anhydrous ether (97:3, *v*/*v*) was selected as the elution solution.

### 2.3. Optimization of the LC-MS/MS Conditions

The choice of the mobile phase in high-performance LC-MS/MS is essential. We compared the effects of methanol–water and methanol (0.01% formic acid + 2.5 mmol/L ammonium formate)—0.01% formic acid in water solution on the peak shape and retention time of vitamin K_1_. The addition of an optimal amount of formic acid contributed to the symmetry of the peak shape and accelerated the peak time. Therefore, a methanol (0.01% formic acid + 2.5 mmol/L ammonium formate)—0.01% formic acid in water solution was selected for the mobile phase of the study.

**Figure 3:**
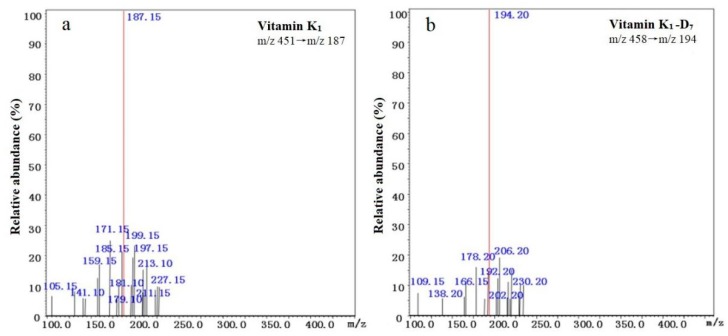
Tandem mass spectra of vitamin K_1_ (**a**) and vitamin K_1_-D_7_ (**b**).

**Figure 4:**
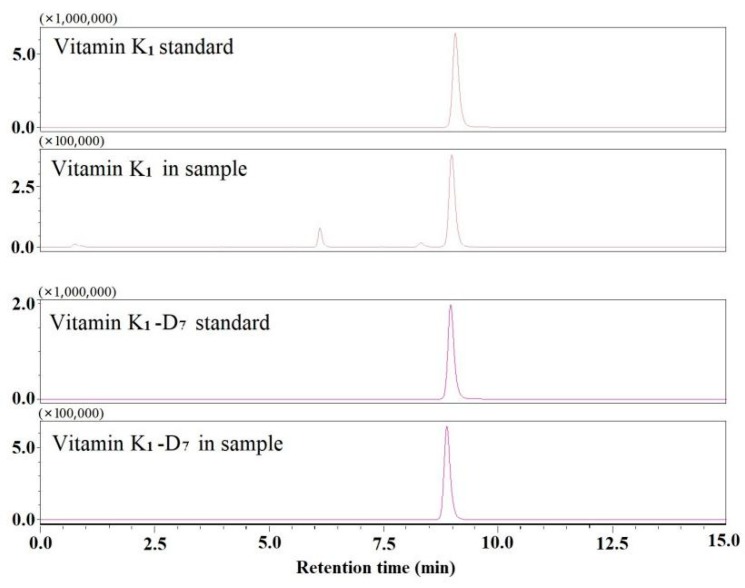
Representative multiple reaction monitoring (MRM) ion chromatograms of vitamin K_1_ and vitamin K_1_-D_7_ in reference standards and samples.

According to tandem mass spectra of vitamin K_1_ and vitamin K_1_-D_7_ (see Figure 3), *m*/*z* 187 and *m*/*z* 194 were selected as quantitative ion for vitamin K_1_ and vitamin K_1_-D_7_, respectively. Flow rate and injection volume can improve the separation of vitamin K_1_. The column pressure, column particle size (1.8 μm), and the optimum flow rate (200–300 μL/min) were optimized for ESI-MS/MS, and set the flow rate to 200 μL/min. The separation effects of 1 and 2 μL were investigated. The K_1_ peak was good when the injection volume was 2 μL, and no leading edge and tailing peak appeared. Therefore, the flow rate used was 200 μL/min, and the injection volume was 2 μL. Chromatograms of vitamin K_1_ and vitamin K_1_-D_7_ are shown in Figure 4.

### 2.4. Method Validation

#### 2.4.1. Linearity, Limit of Detection, and Limit of Quantification

The calibration curve of vitamin K_1_ (see Supplementary Materials) was constructed by using the peak area ratio of five concentrations of vitamin K_1_ standard solutions (triplicates) to the internal standard of vitamin K_1_-D_7_. The limit of detection (LOD) and limit of quantification (LOQ) were evaluated for vitamin K_1_ using a calibration curve based on a signal-to-noise ratio (S/N) of 3 or 10, respectively. The LOD and LOQ were 0.05 and 0.16 µg/kg, respectively. The calibration curve indicated that the excellent linearity was obtained for vitamin K_1_ within a range of 10–500 ng/mL. A typical regression equation was obtained with a correlation coefficient of 0.9988 (Table 1).

#### 2.4.2. Precision and Accuracy

The precision of the method was evaluated by repeated measurements. Intra-day and inter-day precisions were both tested five times by this method under the optimized conditions, and the results were recorded as the relative standard deviation (RSD). The intra-day and inter-day precisions were 1.6–7.2% (Table 2). The accuracy of this method was determined based on the recoveries by adding the standard solutions at three different concentrations (10, 50, and 100 µg/kg) in samples. The recoveries were calculated by comparing the total measured value of the spiked sample with the measured value of the sample, and the ratio of the difference to the spiked amount was the recovery. Recoveries ranged from 80.9% to 119.1% (Table 2). These results showed that the method had high precision, sensitivity, and accuracy for analyzing vitamin K_1_ in fat-containing foods. Compared with previous studies (Table 3), the method used in this study was more sensitive and efficient for detecting vitamin K_1_ in fat-containing foods.

### 2.5. Real Sample Analysis

**Figure 5:**
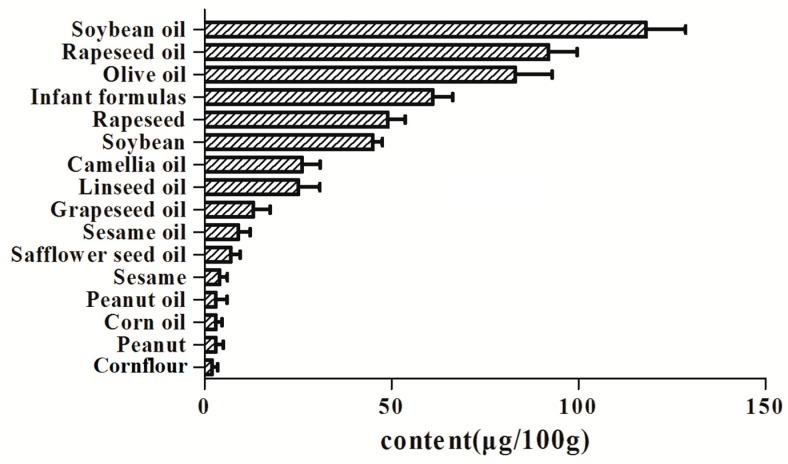
Vitamin K_1_ content in fat-containing foods.

This method is used to determine the content of vitamin K_1_ in fat-containing foods, including rapeseed, soybean, peanut, sesame, corn flour, milk powder, corn oil, peanut oil, safflower seed oil, sesame oil, grapeseed oil, linseed oil, camellia oil, rapeseed oil, soybean oil, and olive oil (see Figure 5). Among them, the content of vitamin K_1_ in soybean oil and rapeseed oil is relatively high; the content of vitamin K_1_ in corn flour and peanut is relatively low. The content of vitamin K_1_ in soybean oil, rapeseed oil, and olive oil summarized by Rebufa [35] was about 105–325 μg/100 g, 70–350 μg/100 g, and 12–100 μg/100 g, respectively (estimated from the picture in the paper). In this study, the average content of vitamin K_1_ in soybean oil, rapeseed oil and olive oil was 118 μg/100 g, 92 μg/100 g, and 83 μg/100 g, respectively. The content of vitamin K_1_ in olive oil and peanut oil determined by Woollard [36] was 93.6 μg/100 g and 1.6 μg/100 g, respectively. In this study, the average content of vitamin K_1_ in olive oil and peanut oil was 92 μg/100 g and 3 μg/100 g, respectively. The comparison indicated that the similar results were obtained in this study with the previous results.

## 3. Materials and Methods

### 3.1. Materials and Reagents

Oilseeds such as rapeseed, soybean, peanut, and sesame were collected. After removing broken, mildew seeds and foreign matters, these samples were pulverized using a grinder and refrigerated them at 4 °C until further processing. Corn flour, infant formulas, and edible oils including soybean oils, rapeseed oils, olive oils, safflower seed oils, Camellia oils, linseed oils, grapeseed oils, sesame oils, peanut oils, and corn oils, were purchased from local supermarkets.

Vitamin K_1_ and vitamin K_1_-D_7_ were purchased from the Toronto Research Chemicals (Ontario, Canada). Methanol and n-hexane (HPLC grade) were purchased from Thermo Fisher Scientific Technology Co., Ltd. (Shanghai, China). Formic acid (98% purity) and ammonium formate (98% purity) were purchased from Aladdin Biochemical Technology Co., Ltd. (Shanghai, China). Anhydrous ether (99.8% purity) was purchased from Sinopharm Chemical Reagent Co., Ltd. (Shanghai, China). Nitrogen (99.99% purity) was purchased from Minghui Gas Technology Co., Ltd. (Wuhan, China). Ultra-pure water (18 mΩ) was obtained from a Milli-Q water purification system (Millipore Co., Ltd., Milford, MA, USA) and was used to prepare all aqueous solutions.

### 3.2. Instruments and Equipment

Analyses were performed on LS-MS/MS analyses with a Shimadzu LC-30AD system coupled with a Shimadzu MS-8060 mass spectrometer (Kyoto, Japan). ZORBAX SB-C18 column (50 mm × 2.1 mm × 1.8 µm) was purchased from Agilent (Santa Clara, CA, USA). CPA224S electronic analytical balance was purchased from Sartorius Company (Göttingen, Germany). The refrigerator was obtained from Siemens (Munich, Germany). The HITACHI CT6E centrifuge was purchased from Hitachi, Ltd. (Tokyo, Japan). The 80350-CN grinder was supplied by Hamilton Beach Electric Co., Ltd. (Shenzhen, China). A DFT-100A portable high-speed universal crusher was purchased from Linda Machinery Co., Ltd. (Wenling, China). The HQ-60 vortex mixer was purchased from North Tongzheng Biotechnology Co., Ltd. (Beijing, China). The DTC-27J ultrasonic cleaner was provided by Dingtai Biochemical Technology Equipment Manufacturing Co., Ltd. (Wuhan, China). SPE column (Mega BE-SI, 1 g, 6 mL) was purchased from Agilent (Santa Clara, CA, USA). Pipettes (10 µL, 200 µL, 1 mL, 5 mL) were purchased from Eppendorf Company (Hamburg, Germany). A 1 mL syringe was purchased from Wangguan Medical Devices Co., Ltd. (Wuhan, China). A 0.22 µm organic phase filter was purchased in Millipore Company (Burlington, MA, USA).

### 3.3. Preparation of Standard Solutions

A stock solution containing 100 μg/mL vitamin K_1_ in methanol was prepared in a brown volumetric flask. A stock solution containing 100 μg/mL of vitamin K_1_-D_7_ internal standard (IS) was prepared. The working standard solutions were prepared by diluting the standard stock solution with methanol. The concentration in the solution for injection was 0.10 μg/mL (IS) for all standard and samples. Calibration standards were prepared in from one stock solution of vitamin K_1_ at concentrations of 0.01, 0.05, 0.10, 0.25, and 0.50 μg/mL. All prepared solutions were stored at −20 °C in darkness.

### 3.4. Sample Preparation

Since vitamin K_1_ is sensitive to light, sample preparation was carried out under low light intensity conditions to minimize photodegradation. A 0.1 g sample (accurate to 0.001 g) was weighed and placed it in a 10 mL tube. All test portions were spiked with 0.1 mL of a 1 μg/mL IS solution. Then, 7 mL of n-hexane was added to each test tube and vortexed the solution to ensure adequate mixing. The purpose of the ultrasound-assisted extraction was to increase the fluidity of the sample and to allow the target to be rapidly and completely transferred to the extractant [20,21]. After single factor experiments, the sample in the test tube was subjected to ultrasonic-assisted extraction for 50 min in an ultrasonic generator with an ultrasonic power of 700 W and a temperature of 50 °C. The sample was then centrifuged at 4500 r/min for 6 min. The upper hexane layer was transferred to a new tube for purification.

Solid-phase extraction (SPE) was used to remove lipids and lipophilic pigments from the extracts. The SPE silica gel column was first activated with 6 mL of n-hexane, and then the extract was passed through the column at a flow rate of 1.0 mL/min. The cartridge was rinsed with 6 mL of n-hexane at a flow rate of 1.0 mL/min. Finally, 8 mL of a solution of n-hexane/diethyl ether (97:3, *v*/*v*) was added and the eluate was collected. After the eluent was dried with nitrogen, 1 mL of methanol was added, followed by vortexing. After filtration through a 0.22 μm organic filter, a 2 μL sample was injected into LC-MS instrument for analysis.

### 3.5. LC-MS/MS Method

A C18 column (ZORBAX SB-C18, 50 mm × 2.1 mm, i.d. 1.8 μm, Agilent) was used to isolate vitamin K_1_ at 40 °C. The injection volume for the sample was 2 μL by an automatic sampler. Mobile phase B was methanol with 0.01% formic acid and 2.5 mmol/L ammonium formate (*v*/*v*). Mobile phase A was water with 0.01% formic acid (*v*/*v*) at a flow rate of 0.2 mL/min. Gradient program was as follows: 0–2 min 70% B; 2–14 min from 70% to 98% B; 14–14.1 min from 98% to 70% B; 14.1–15 min from 70% to 70% B.

The multiple reaction monitoring (MRM) transitions were optimized by direct infusion of compounds from a standard solution containing 1 μg/mL in an automatic mode. The collision voltage and the appropriate atomization gas flow rate and drying gas flow rate were optimized. Mass spectrometric conditions were optimized as follows: DL temperature 250 °C, heat block temperature 400 °C, nebulizing gas 3 L/min (N_2_), and drying gas 10 L/min (N_2_). The MS/MS parameters for the analysis of compounds are shown in Table 4. Tandem mass spectra of vitamin K_1_ and vitamin K_1_-D_7_ are shown in Figure 3. All determinations were performed in triplicate.

### 3.6. Peak Identification

In the multiple reaction monitoring mode, the peak of the target in the sample was determined based on the retention time (RT) and the mass spectrum of the vitamin K_1_ standard.

### 3.7. Statistical Analysis

All results were expressed as average values with three replicates per sample. The measurements were completed with an internal standard correction curve method. Data acquisition and processing were controlled using LabSolutions software (Kyoto, Japan). Statistical analyses were performed with Microsoft Office 2010 (Redmond, WA, USA) and GraphPad Prism software (San Diego, CA, USA).

## 4. Conclusions

An efficient, sensitive, and reliable method was developed to analyze vitamin K_1_ in fat-containing foods. The method used ultrasound-assisted extraction, SPE column purification treatment, and high-performance LC-MS/MS. This method had favorable linearity, good recoveries, good intra-day and inter-day precisions, and low LOD and LOQ. Compared with the enzymatic hydrolysis method in the national standard, the method established in this paper has a simple pretreatment process, significant substrate purification effect, and accurate and reliable detection results. Based on this method, the contents of vitamin K_1_ in 16 fat-containing foods such as rapeseed, soybean, and olive oil were successfully quantified. The content of vitamin K_1_ in edible oil was significantly higher than that in oilseeds and other fat-containing foods. Among the tested samples, the lowest content of vitamin K_1_ was corn flour, and the highest content was soybean oil. It is hoped that the above results can promote the improvement of nutrient utilization in oil-containing foods, strengthen research on high-vitamin K_1_ oil-containing foods, and promote the healthy development of the food industry.

## Figures and Tables

**Table 1:** Linear equation, determination coefficients, LOD and LOQ for vitamin K_1_.

Compound	Calibration Curve	Linear Range (µg/kg)	*R* ^2^	LOD (µg/kg)	LOQ (µg/kg)
Vitamin K_1_	Y = 1.4831X − 0.1171	10–500	0.9988	0.05	0.16

**Table 2:** Precision and accuracy of the method.

Compound	Spiked Concentration (μg/kg)	Intra-Day Precisions (RSD%, *n* = 5)	Inter-Day Precisions (RSD%, *n* = 5)	Recovery (%, *n* = 3)
Vitamin K_1_	10	3.7	6.2	93.2/119.1/107.1
	50	1.6	4.3	91.0/89.3/89.1
	100	5.4	7.2	80.9/82.8/81.5

**Table 3:** Comparison of the method proposed in this study with the previous studies.

No	Sample	Sample Pretreatment	Determination Technique	Column	LOQ	Recovery (%)	Analysis Time (min)	Literatures
1	Corn oil	Enzymatic digestion and extraction	HPLC-Fluorescence	YMC C30 Wilmington (250 × 4.6 mm, 3 μm)	-	82~100	30	[14]
2	Spinach, peas, apples, et. al	ASE-SPE	HPLC-MS/MS (APCI)	Phenomenex Kinetex PFP (100 × 2.1 mm, 2.6 μm)	0.5 μg/100 g	90~120	20	[17]
3	Fruits and vegetables	LLE-SPE	LC-MS/MS (ESI)	ZORBAX Eclipse plus C18 (50× 2.1 mm, 1.8 μm)	0.004 mg/kg	84~115.6	8	[30]
4	Rapeseed or Canola oil	Enzymatic hydrolysis and SPE	HPLC-Fluorescence	PartiSphere Whatman C18 (150 × 4.6 mm, 6 μm)	-	80.8~95.4	30	[31]
5	Fermented food	SPE	HPLC-MS/MS (APCI)	Phenomenex Kinetex C18 (100 × 2.1 mm, 1.7 µm)	0.385 ng/g	99.8	10	[32]
6	Rice bran and vegetable oil	LLE	HPLC-DAD-FLD	Phenomenex Kinetex PFP (250 × 4.6 mm, 5 μm)	0.915 μg/mL	96.0~102.9	30	[33]
7	Cooking oils	LLE	UPC2	HSS C18 SB (100 × 3.0 mm, 1.8 μm)	0.98 μg/mL	94.24 ± 0.56	8	[34]
8	Fat-containing foods	UAE-SPE	HPLC-MS/MS (ESI)	ZORBAX SB-C18 (50 × 2.1 mm, 1.8 μm)	0.16 μg/kg	80.9~119.1	15	This study

**Table 4:** MS/MS parameters for analysis of compounds.

Compounds	Scan Mode	RT (min)	Parent Ions (*m*/*z*)	Product Ions (*m*/*z*)	Collision Energy (eV)
Vitamin K_1_	[M + H]^+^	9.058	451	227/187 *	23/25
Vitamin K_1_-D_7_	[M + H]^+^	8.980	458	194*	18

* indicate: quantitative ion.

## Supplementary Materials

The following are available online at https://www.mdpi.com/1420-3049/25/4/839/s1.
