# Supplementary material for: Extraction and Determination of Vitamin K1 in Foods by Ultrasound-Assisted Extraction, SPE, and LC-MS/MS
Source: Molecules. 2020 Feb 14;25(4):839. doi: 10.3390/molecules25040839 (PMC7070738; doi:10.3390/molecules25040839)

Supplementary materials

Figure S1. Calibration curve of vitamin K_1

Figure S2. MS/MS spectrum of vitamin K_1 and vitamin K_1-D_7: (a) vitamin K_1 full scan, (b) vitamin K_1 product ion scan, (c) vitamin K_1-D_7 full scan, (d) vitamin K_1-D_7 product ion scan

Figure S1. Calibration curve of vitamin K_1

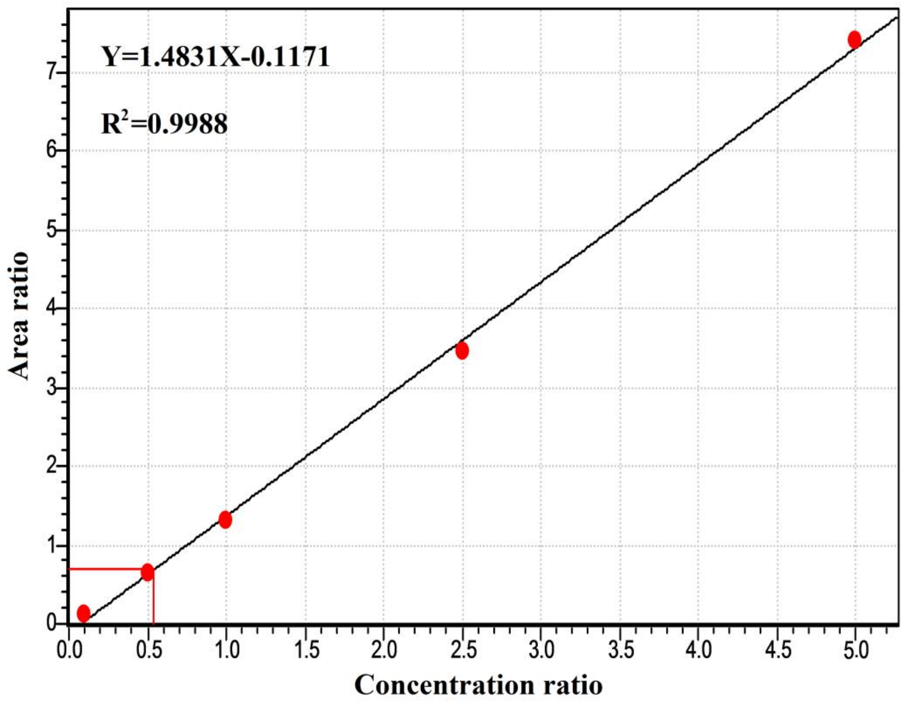

Figure S2. MS/MS spectrum of vitamin K_1 and vitamin K_1-D_7: (a) vitamin K_1 full scan, (b) vitamin K_1 product ion scan, (c) vitamin K_1-D_7 full scan, (d) vitamin K_1-D_7 product ion scan

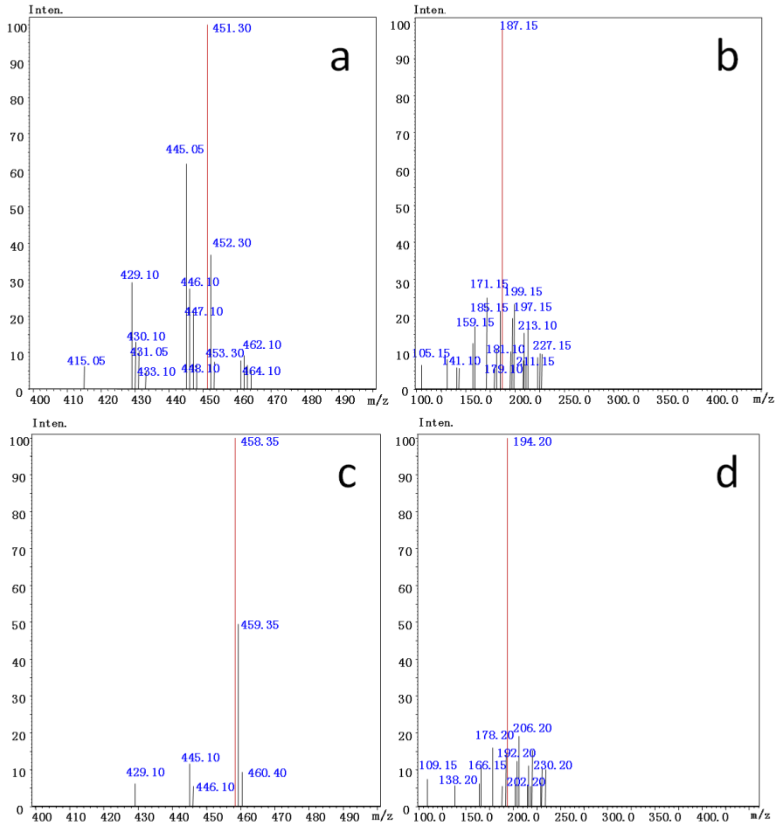

Supplement: Supplementary file 1 [file molecules-25-00839-s001.pdf]
